# Barriers to and Facilitators of Using a One Button Tracker and Web-Based Data Analytics Tool for Personal Science: Exploratory Study

**DOI:** 10.2196/32704

**Published:** 2022-03-01

**Authors:** Tom H van de Belt, Aimee de Croon, Faye Freriks, Thomas Blomseth Christiansen, Jakob Eg Larsen, Martijn de Groot

**Affiliations:** 1 Health Innovation Labs Radboud University Medical Center Nijmegen Netherlands; 2 Konsulent Blomseth Copenhagen Denmark; 3 Danmarks Tekniske Universitet Compute (DTU) Technical University of Denmark Copenhagen Denmark

**Keywords:** self-tracking, personal science, one-button-tracker, barriers, facilitators, quantified self, health promotion, button tracker, usability testing, One Button Tracker, health technology, system usability

## Abstract

**Background:**

Individuals’ self-tracking of subjectively experienced phenomena related to health can be challenging, as current options for instrumentation often involve too much effort in the moment or rely on retrospective self-reporting, which is likely to impair accuracy and compliance.

**Objective:**

This study aims to assess the usability and perceived usefulness of low-effort, in-the-moment self-tracking using simple instrumentation and to establish the amount of support needed when using this approach.

**Methods:**

In this exploratory study, the One Button Tracker—a press-button device that records time stamps and durations of button presses—was used for self-tracking. A total of 13 employees of an academic medical center chose a personal research question and used the One Button Tracker to actively track specific subjectively experienced phenomena for 2 to 4 weeks. To assess usability and usefulness, we combined qualitative data from semistructured interviews with quantitative results from the System Usability Scale.

**Results:**

In total, 29 barriers and 15 facilitators for using the One Button Tracker were found. Ease of use was the most frequently mentioned facilitator. The One Button Tracker’s usability received a median System Usability Scale score of 75.0 (IQR 42.50), which is considered as good usability. Participants experienced effects such as an increased awareness of the tracked phenomenon, a confirmation of personal knowledge, a gain of insight, and behavior change. Support and guidance during all stages of the self-tracking process were judged as valuable.

**Conclusions:**

The low-effort, in-the-moment self-tracking of subjectively experienced phenomena has been shown to support personal knowledge gain and health behavior change for people with an interest in health promotion. After addressing barriers and formally validating the collected data, self-tracking devices may well be helpful for additional user types or health questions.

## Introduction

### Personal Science

An increasing number of people collect data on personal health or lifestyle phenomena, such as physical activity levels, mood, or sleep quality, for the purpose of self-reflection or personal knowledge gain. This practice of *self-tracking* or *self-quantification* can be supported with technological instruments such as wearable devices or mobile apps [1]. Enhancing personal knowledge supports health maintenance and may facilitate behavioral change [2-4]*.* In a clinical context, self-tracked data may be used for shared decision-making because it can improve communication, enhance bilateral coordination of care, and boost patient engagement and sense of autonomy [5-7]. Effective self-tracking not only mediates personal health behavior but also holds promise as a complementary field of knowledge creation and discovery. As a data acquisition method, self-tracking is integral to participant-led research (PLR) and personal science [8-11]. In PLR and personal science, patients or participants transcend their traditional role as a source of data by initiating and conducting research projects themselves. This includes acquisition and reflection of personally relevant data by and for themselves. Such an active role of a patient or citizen leading their own research is what defines and also connects PLR and personal science.

### Self-tracking

In self-tracking, objective measures of an increasing range of physiological and behavioral phenomena can be automatically and accurately recorded by wearable sensors incorporated in activity trackers, smart watches, or other self-tracking devices. However, tracking subjectively experienced phenomena that manifest as physical sensations such as different moods, stress, discomfort, pain, mental flow, thoughts, emotions, or social interaction remains challenging. Instruments aimed at tracking these subjective phenomena as a primary or secondary outcome measurement using empirical methods typically involve diary entry or experience sampling methods with prompted self-reports, which can lead to inaccurate data due to associated memory recall biases [12-14]. Alternative options for in-the-moment registration, such as paper-based tracking, involve high effort, which may impair sustained use. These barriers limit discovery using self-tracking of subjectively experienced phenomena. To overcome these barriers, self-tracking devices that facilitate low-effort, in-the-moment tracking have attracted research interest. A pilot study exploring the use of a smart button device for the purpose of self-tracking medication adherence found that participants generally considered the device acceptable to use, but the collected data had poor concordance with electronic data collection [15].

### One Button Tracker and Objectives

In recent case studies, the use of One Button Tracker instrumentation was introduced to facilitate low-effort, in-the-moment self-tracking [16,17]. The One Button Tracker is a data acquisition instrument that allows users to track any subjectively experienced phenomenon in the moment it occurs with little effort by a push of the single button. The point in time and duration of the button press are recorded, and the acquired data points can be loaded into a web-based data analytics tool. There, the collected data are automatically displayed in a calendar overview and graphs showing hourly and weekly distribution of observations. In one study [16], a patient with posttraumatic stress disorder was able to learn about the nature of his symptoms and the conditions in which they would arise by tracking a subjectively experienced precursor to one of his symptoms. In another study [17], the One Button Tracker enabled the investigation of temporal dynamics of and relations between two different subjectively experienced symptoms (intrusions and ruminations related to posttraumatic stress disorder). These studies suggest that the One Button Tracker in combination with a web-based data analytics tool can support low-effort, in-the-moment self-tracking. However, this promise remains a matter of research. Therefore, this exploratory study has two aims: to assess the usability and perceived usefulness of the One Button Tracker as instrumentation and to establish the amount of support needed using this approach.

## Methods

### Study Design and Setting

In this pilot study, we used a mixed methods approach to assess the usability and perceived usefulness of low-effort, in-the-moment tracking using simple instrumentation and to evaluate the support needed to allow individuals to self-track effectively. The qualitative part of the study consisted of semistructured interviews exploring facilitators and barriers to the use of this self-tracking method, perceived positive and negative effects of its use, and participant views on the potential value of support during the self-tracking process. The quantitative data were generated through a usability survey. Data were collected from February to August 2020. Ethical approval was granted by the local medical ethics committee (Medisch Ethische Toetsingscommissie Oost-Nederland, review number 2019-6066) and standards for reporting qualitative research were followed [18]. All participants provided written informed consent before participating in the study.

### Participants

Participants were recruited from an existing cohort of employees from Radboud University Medical Center (Radboudumc, Nijmegen, the Netherlands) enrolled in the hospital’s health promotion program *Healthy Professionals*. This program strives to educate employees on health and lifestyle topics to help them remain resilient and healthy in the rapidly changing health care environment [19]. This study was embedded within the Healthy Professionals program as self-tracking may aid participants in working toward their formulated lifestyle goals and because of the pioneering character of the program, which suited the explorative character of this study well. All 101 health care professionals enrolled in the program at the time of recruitment (May 2020) were invited to participate in the study by email. If they expressed interest, a researcher (AC) contacted them by phone to determine eligibility and answer any questions regarding the study’s protocol. Applicants were eligible if they were aged ≥18 years. They were excluded if they were not able to verbally communicate in Dutch or had cognitive dysfunction. We aimed to include 12-15 participants, which was deemed as sufficient for this exploratory study.

### The One Button Tracker

**Figure 1:**
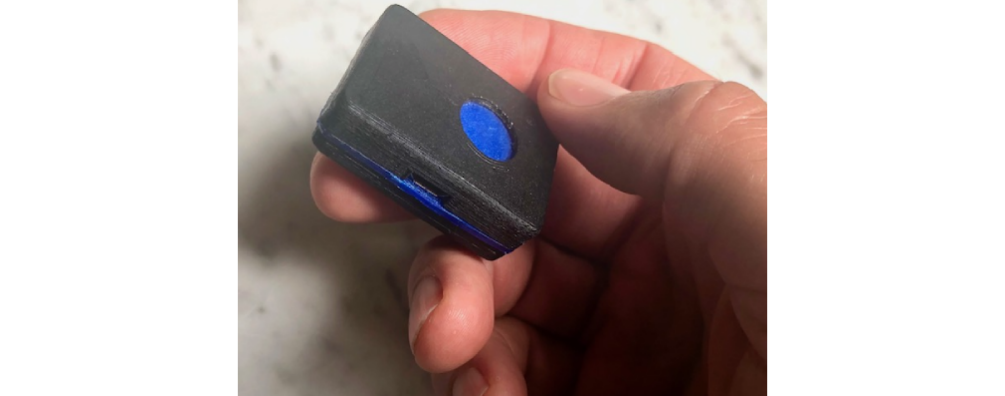
One Button Tracker. It is a data acquisition instrument in a 3D printed plastic casing that can be charged via a USB port. It was designed to track any subjectively experienced phenomenon.

For this study, we used a research prototype of the One Button Tracker, as depicted in Figure 1. The instrument was invented and developed by the coauthors JEL and TBC. The instrument is 41×31×12.5 mm in size, consists of a low-powered (3.3 V) press-button tracker within a 3D printed plastic casing, and can be charged via a USB port. When pressed, the device vibrates, thereby providing haptic feedback to the user. The point in time and duration of the button press were recorded. In the processing of the acquired data, these attributes can be used to distinguish between single, double, or more presses and shorter and longer durations of the presses, which can be used for different purposes depending on the user’s needs. The One Button Tracker has no wireless or internet connection to ensure privacy, and the user is in control of the acquired data. The time required for a user to record an observation using the instrument was <1 second.

Data stored on the device can be accessed by connecting the One Button Tracker to a laptop or desktop computer via a USB connection. The stored data file containing timestamps and button press durations can be loaded into a web-based data analytics tool. There, the collected data are automatically displayed in an overview table. In addition, several graphs are created that portray the average number of button presses per hour, day, week, and month (examples of visualizations from the data analytics tool are shown in Multimedia Appendix 1).

### Study Procedures

Participants were sent instructions on the use of the One Button Tracker and web-based data analytics tools by email. These detailed how to operate the device; explained its ability to distinguish between 1, 2, and 3 presses; and instructed participants to charge the device twice a week. In addition, the hyperlink to the web-based data analytics tool was provided, accompanied by written instructions in a step-by-step manner on how to transfer data to the tool. In an intake session, these instructions were restated, and remaining questions were answered.

Thereafter, participants worked toward a suitable personal research question with the help of a researcher (AC). If participants had already formulated questions related to their health promotion goals before the Healthy Professionals intake session, these questions were examined to determine their suitability. If formulating a question was revealed to be difficult, the researcher used open-ended questions to explore the knowledge gaps in the path toward their formulated health goals. A research question was deemed suitable if it related to the participants’ set health or lifestyle goals, was personally relevant, and was answerable by self-tracking a given phenomenon. Participants were strongly advised to pick a phenomenon that would result in ≥2 and ≤20 clicks per day to avoid tracking fatigue.

Subsequently, participants started the self-tracking process in which they used the One Button Tracker to actively track the chosen phenomenon for a minimum of 2 and a maximum of 4 weeks, depending on their research question and preferences. During this time, participants could view the collected data through the data analytics tool on their computer when desired. In case of problems, they could contact a technical support helpline by phone or email. In addition, we performed a weekly checkup by phone to answer any questions and gauge the use of the device. Any remarks regarding the One Button Tracker or the self-tracking process participants made in these calls were noted and added to the record for analysis.

**Figure 2:**
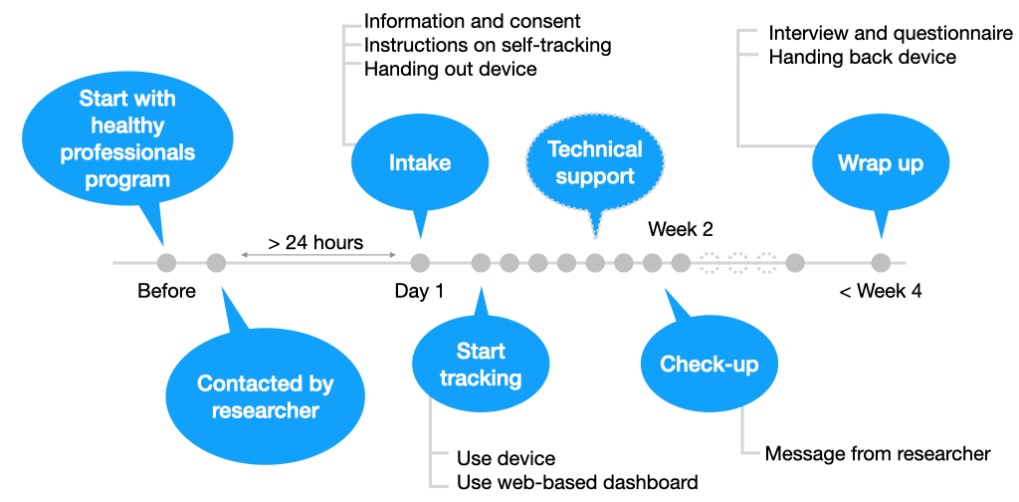
Overview of the study’s timeline. Self-tracking was carried out by the participants for a duration of 2 to 4 weeks.

When participants finished tracking, they handed the device back to the researchers. In individual interviews, their experiences using the One Button Tracker and doing self-tracking were explored. In these sessions, the data collected by the participants were loaded into the web-based data analytics tool and discussed. Afterward, all data were deleted permanently. Following the interview, participants were asked to fill out the System Usability Scale (SUS) questionnaire to determine the usability of the One Button Tracker. Figure 2 provides an overview of the study’s timeline.

### Data Collection

All semistructured interviews were conducted in July 2020 by a junior researcher (AC) with previous training in qualitative research. The interview guide was developed specifically for this study, drawing from insights from Li et al [20] and Almalki et al [21] regarding personal informatics systems. It included questions on participant expectations, their personal research question and approach, views of and experiences with the One Button Tracker and the web-based data analytics tool, and views of and experiences with self-tracking and support during this process (Multimedia Appendix 2). All interview questions were open-ended. In addition to these questions, participants were asked to grade the OBT on a scale from 1 (worst possible functioning) to 10 (best possible functioning).

During the interview sessions, we also collected demographic information, including birth year, education, and job description. We initially intended to interview all participants face-to-face at the participants’ location of choice. However, owing to COVID-19 restrictions, half of the interviews were conducted via the internet using Skype for Business (version 7.0.2676.1, 2018; Microsoft Corporation). All interviews were audio-recorded, transcribed clean verbatim, and anonymized.

Following the interview, participants completed a Dutch version of the SUS [22] (Multimedia Appendix 3). This short 10-item validated questionnaire is widely used to determine the usability of devices or systems. It asks participants to rate the truthiness of 10 statements concerning the device’s usability on a 5-point Likert scale.

### Data Analysis

A total of 2 researchers (AC and FF) analyzed the anonymized transcripts independently using qualitative data analysis software (ATLAS.ti version 7.1; Scientific Software Development GmbH). They identified barriers, facilitators, and positive and negative effects of the use of the One Button Tracker for self-tracking. The identified codes were thoroughly discussed until consensus was reached. Remaining disagreements were discussed with a third researcher (TB). To determine if the sample size was sufficient to gain a comprehensive overview of participant experiences, code saturation was assessed after each third interview by examining whether any previously unnamed barriers and facilitators or effects were identified in the newly gathered data. We defined saturation as 3 subsequent interviews with no new factors.

Facilitators and barriers regarding the use of the One Button Tracker were categorized according to the Gagnon framework concerning determinants of adoption of information and communications technologies in health [23,24]. New barriers and facilitators were added to the framework. The Donabedian framework for the quality of health care was used to present all identified positive and negative effects [25]. This framework distinguishes structure (context in which health care is delivered), process (all actions that make up health care), and outcome (all effects on patients’ health). Dutch quotes and themes used in this paper were translated into English and checked by all authors.

Participants’ answers on the SUS were computed as described by Brooke et al [22], resulting in a score between 0 and 100. Scores were interpreted in accordance to Bangor et al [26], where with a score of ≤50.9, the usability of a device is deemed *poor*; with a score of >50.9, usability is deemed *sufficient*; with a score of >71.4, usability is deemed *good*; with a score of >85.5, usability is deemed *excellent*; and with a score of >90.9, usability is deemed the *best imaginable*.

Analysis and statistics were performed in the Radboudumc using SPSS (version 25.0; IBM). Normally distributed continuous variables are described using mean and SD. Median and interquartile values were shown in case variables were not normally distributed. Qualitative or categorical variables were described using frequencies and percentages.

### Data Storage and Privacy

After each interview, the pseudonymized audio record was stored within the Radboudumc data storage environment until transcription was finished, in accordance with Dutch privacy law. After transcription and coding, the anonymized transcripts were archived according to the Radboudumc research policy.

## Results

### Participants

Of the 101 professionals who were invited to participate, 58.4% (59/101) actively declined. Provided reasons for not participating included no time, increased workload due to the COVID-19 pandemic (7/59, 12%) already able to reach health goals (3/59, 5%), not wanting to use this particular self-quantification system (3/59, 5%), changing jobs (2/59, 2%), and illness (2/59, 2%). A total of 13 professionals expressed interest and were included, resulting in a recruitment rate of 12.8% (13/101). Table 1 provides an overview of all participants’ characteristics.

**Table 1:** Participant characteristics (n=13).

Characteristic				Value
**Gender, n** **(%)**				
	Female		11 (85)	
	Male		2 (15)	
Age (years), median (range)				56 (35-67)
**Educational background, n (%)**				
	Vocational		3 (23)	
	Applied sciences		6 (46)	
	Academic		4 (31)	
**Job type, n (%)**				
	Management		7 (54)	
	**Medical staff**			
		Nursing and care	3 (23)	
		Medical doctor and specialists	0 (0)	
	Staff, administration, or secretary		3 (23)	

### Self-tracking

All participants successfully formulated a personal research question and used the One Button Tracker for self-tracking. A total of 85% (11/13) of the participants made use of the device’s ability to distinguish between 1, 2, and 3 button presses. Some used this feature to indicate the intensity of the tracked phenomenon, where more presses indicated a stronger or more intense experience, whereas others assigned different relevant phenomena to different numbers of presses to explore their relationships. Table 2 provides an overview of all personal research questions and tracked phenomena. When participants were asked to restate their formulated personal question during the interviews, they often posed their focus as a behavioral goal or an aim, rather than as a (research) question.

**Table 2:** Topic, question, and description of the self-tracked phenomenon of each of the 13 participants’ personal research projects.

ID	Topic	Personal research question	Phenomenon description
01	Creativity	How can I identify and facilitate creative moments in my day-to-day work?	1 press=creative moment; 2 or 3 presses=more intense experience
02	Snacking	How often do I snack?	1 press=snack
03	Stress	What factors influence my (experience of) heart palpitations?	1 press=palpitations; 2 or 3 presses=more intense experience (used diary to record contextual factors)
04	Stress	To what extent do I experience physical stress symptoms?	1 press=muscle tension; 2 presses=tensed breathing; 3 presses=cannot eat
05	Stress	What is for me the relation between stress and movement?	1 press=stress; 2 presses=moving a little; 3 presses=moving a lot
06	Hydration	How much do I drink and how does this relate to thirst?	1 press=thirst; 2 presses=drinking
07	Creativity	How can I get out of a rut, using music?	1 press=effective music-based intervention
08	Hydration	How much do I drink and how does this influence my headaches?	1 press=drinking; 2 presses=headache; 3 presses=pain killers
09	Drinking	How much coffee and wine do I consume?	1 press=cup of coffee; 2 presses=glass of wine
10	Wellness	At what times do I feel energetic?	More presses=feeling better; fewer or no presses=feeling worse
11	Disquiet and food	What is for me the relationship between mental unrest and thinking of food?	1 press=thinking of food; 2 presses=mental unrest
12	Fidgeting	Does my fidgeting habit follow a recognizable pattern, and how might knowledge of this pattern help me to fidget less?	1 press=fidgeting with fingers; 2 presses=touching face
13	Hydration and stillness	How much do I drink, and how can I intercept my working day with mindfulness exercises to improve stillness?	1 press=drinking; 2 presses=mindfulness exercise

The duration of the self-tracking projects ranged from 6 to 38 days, with a median of 23. Of the 13 participants, 3 (23%) deviated from the self-tracking period of 2 to 4 weeks. From these 13 participants, 1 (8%) ended the tracking project after 8 days because of a perceived lack of usefulness and high burden, another (1/13, 8%) ended the project after 6 days because she had answered her personal research question, and the last (1/13, 8%) extended the tracking period because of illness. During the tracking period, 54% (7/13) of the participants visualized the collected data using the web-based data analytics tool. Moreover, of the 13 participants, 2 (15%) used the tool for data interpretation, whereas the other 5 (38%) used it to check whether the One Button Tracker was still fully operational. Of the 6 participants who did not successfully load their data into the web-based tool, 1 (17%) intended to but encountered technical issues, whereas the others had no interest in viewing the collected data.

### Technical Challenges

Of the 13 participants, 6 (46%) participants encountered technical difficulties during the tracking period. After troubleshooting, it turned out that a technical error had resulted in the loss of all software and data files. In 3 cases, no personal data were lost, as the technical error had occurred before self-tracking commencement. However, 23% (3/13) of the participants lost (a proportion of) their collected data when this transpired.

Multiple possible causes for this issue were identified. Of the 13 participants, 1 (8%) had accidently connected the One Button Tracker to the hospital’s computers, which had been warned against in the written instructions, as this was known to elicit a faulty reset of the One Button Tracker. Another (1/13, 8%) participant inadvertently caused an error by incorrectly detaching the One Button Tracker from their PC. In other cases, the cause of the errors remained unclear. After consultation with the coauthors JEL and TBC, all One Button Trackers received a software update. From there on, no further technical issues with the One Button Trackers were encountered.

### Qualitative Results

The interviews ranged from 15 to 56 minutes in length. Analysis of the interviews revealed 29 barriers and 15 facilitators, as well as 2 negatively and 12 positively perceived effects. Textbox 1 presents all identified barriers and facilitators. Textbox 2 presents an overview of the experienced effects. Data saturation could not be confirmed.

**Barriers to and facilitators regarding the use of the One Button Tracker as a self-tracking instrument according to the Gagnon framework of determinants of adoption of information and communications technologies in health care. Barriers and facilitators are ordered in three categories (technological, individual, and external), including the number of participants who mentioned each identified barrier (B, n) or facilitator (F, n).**

**Technological barriers and facilitators related to mobile health characteristics**

1. Design and technical concerns
  Unattractiveness of design (B, 1)
  Physical aspect of the device reminds user to track (F, 1)
  Data analytics tool’s visualizations are attractive (F, 1)
  Device is too small to be aware of (B, 1)
  Device’s button (cannot be pressed or [lack of] confirmation; B, 1)
  Device’s button (vibrations assure user of correct press; F, 1)
  Device is not compatible with operating system (B, 1)
  Disfunction not further specified (B, 1)
2. Perceived usefulness
  Set goal is personally important to user (F, 1)
  Lack of usefulness after research question has been answered (B, 1)
  No need to use device or dashboard (B, 4)
  Lack of confidence that research question will be answered (B, 1)
3. Perceived ease of use
  Device is handy (F, 3)
  Device is easy to use (F, 5)
  Carrying device is a bother: already have to bring a smartphone (B, 1)
  Challenging to transport the device, especially when the user has no pockets (B, 5)
  Visualizing the data in the data analytics tool involves too much effort (B, 3)
  User needs instructions on how to interpret the data analytics tool’s visualizations (B, 2)
  Fear to lose device (B, 1)
  Psychological stress of using the device is zero, which facilitates use (F, 1)
4. Privacy and security concerns
  Data cannot be removed accidently (F, 1)
  Good privacy protection when compared with other tools such as app (F, 1)
5. Satisfaction about content available (completeness)
  Lack of in-the-moment feedback (B, 1)
  Hard to determine when to use the device specifically with subjective phenomena (B, 1)
6. Content appropriate for users (relevance)
  Sense of failure each time a negatively judged phenomena is tracked (B, 1)
7. Accuracy
  User forgets to bring the device when on the move (B, 7)
  User forgets to track when device is out of sight (B, 1)
  User forgets to track when distracted by other activities (B, 1)
  Incorrect categorization of measure due to changes midactivity or midexperience (B, 1)
  Button cannot be pressed accidently (F, 1)
  User forgets device when clothes are regularly changed, for example, health care workers (B, 1)
  After a few weeks user forgets to track (B, 1)

**Individual barriers and facilitators: knowledge, attitude, and sociodemographic characteristics**

1. Time issues
  User is too busy to view the data analytics tool (B, 2)
  Interpreting the data analytics tool visualizations is too time-consuming (B, 1)
2. Outcome expectancy
  While working night shifts, life differs so substantially that tracking during these shifts does not lead to generalizable knowledge (B, 1)
3. Agreement with mHealth (welcoming or resistant)
  Tracking is fun (F, 1)
  Not motivated to use data analytics tool (B, 1)

**External barriers and facilitators: social and training environment**

1. Social pressure (associated with peers)
  Device can be used unnoticed by others (F, 1)
  Lack of use when other people are around (B, 1)
  Less comfortable to use device among others: fear of having to explain him or herself or to disturb group progress (B, 1)
2. Training
  Support and guidance during the process of designing a personal research question (F, 1)
  No behavioral goal formally set in collaboration with researcher (B, 1)
3. Communication and collaboration effort
  Regular checkups support user motivation (F, 1)
4. External environment
  Research context elicits motivation (F, 2)

**Positive and negative effects of the use of the One Button Tracker as a self-tracking instrument according to the Donabedian framework for quality of care. Effects are ordered in two categories (process and outcome) including the number of participants who mentioned each positive effect (P, n) or negative effect (N, n).**

**Process**

1. Self-tracking process
  It is enjoyable to consciously focus on a certain experience or behavior (P, 2)
  Tracking causes confrontation with failure in the set goal (N, 1)
  The device causes annoyance (N, 1)
2. Tracked personal data
  Gathered data can be related to lived experiences (P, 3)
  The ability to differ between the number of presses facilitates a differentiated overview of the user’s progress (P, 1)
  Data analytics tool visualizations increase insight into temporal fluctuations in the tracked phenomenon (P, 1)

**Outcome**

1. Self-awareness
  Enhanced awareness of tracked experience or phenomenon (P, 8)
2. Personal knowledge
  (Objective) confirmation of existing beliefs (P, 3)
  Gain of personal insights (P, 4)
  Reassurance that user can take control of own health (P, 1)
3. Action
  User is incentivized to turn goal setting into action (P, 4)
  Behavioral change (P,4)
  The device functions as an incentive to perform the desired behavior (P, 3)
  The device functions as a reinforcement to put the gained insights into practice (P, 3)

We identified a diverse number of barriers and facilitators that influence the uptake of the One Button Tracker. However, 3 aspects of the One Button Tracker’s usability were emphasized by participants. The *user-friendliness of the device* was one such aspect. Because of its small size and the simplicity of its design, the One Button Tracker was generally considered easy to use. Second, most participants thought of the One Button Tracker as *easily portable*, as users can carry the device with them in a trouser or shirt pocket. A third mentioned asset of the One Button Tracker was that its *size facilitates pressing its button unseen by others*, which was considered to be of added value:

I could just quickly press it, use it unnoticed. So I er, I didn’t need to grab some clumsy-looking apparatus, “what have you got there?”. You can just nicely...just quietly press it.

Participant 03

Although collecting personal data with the One Button Tracker was regarded to be convenient, there were certain aspects of the data collection process that participants viewed as possibly troublesome. Although the One Button Tracker was considered to be easily portable, it could be *challenging for users to remember bringing the device* when they were on the move. This effect was said to be more pronounced when participants’ clothing lacked pockets to transport the One Button Tracker. Even when participants brought the One Button Tracker with them, missed tracking points still occurred. Participants explained that they *sometimes forgot to track experiences when distracted by other activities* or when they had placed the One Button Tracker out of their line of sight. Because of these occurrences, of the 13 participants, 5 (38%) reported instances of missed or misregistered data points. However, even with these missed data points, the majority still felt that the collected data accurately represented their experienced reality:

One time, I wasn’t wearing trousers with pockets. And then I realized, oh shoot, forgot it. No trousers, no tracker.

Participant 05

Another factor of which the importance was emphasized is the availability of tailored support during the tracking process. Without guidance, it would be difficult to design a personally relevant research question and to select an appropriate phenomenon to track. Furthermore, some participants felt that the points of contact with the researchers during the weekly checkups facilitated a boost in motivation to persist in the tracking process. Appropriate support was also judged to be essential for correct interpretation of collected data presented in the web-based data analytics tool. Overall, 31% (4/13) of the participants mentioned that they were interested in looking at their personal data but needed support interpreting the data visualizations. Some stated they thought connecting the device to their desktop and loading the collected data involved too much effort or was too time-consuming. All in all, support and guidance during all stages of the self-tracking process were judged as very important:

Well, I did see those graphs. And I did have a look at them, but I thought yeah, I’m going to need some explanation from you here, what you guys think of this.

Participant 03

Participants experienced a range of effects to be the result of the use of the One Button Tracker for self-tracking. Although most of these effects were judged positively, of the 13 participants, 1 (8%) described that she experienced negative emotions throughout the tracking process. This participant had chosen to track a behavioral phenomenon that she wanted to perform less often, which resulted in a sense of failure each time this behavior was registered. As for positive effects, 62% (8/13) of the participants stated that the use of the One Button Tracker led to enhanced awareness of the tracked phenomenon. This facilitated an objective confirmation of existing beliefs but could also lead to the gain of entirely new personal insights. For 31% (4/13) of the participants, enhanced awareness and gained personal knowledge culminated in or contributed to behavioral change. Another important effect mentioned was the functioning of the One Button Tracker as an incentive for desired behavior. Participants explained that the device reminded them or even motivated them to act more in line with their stated behavioral goal:

Seeing that thing laying there, thinking oh right, oh I can do that now! That does have a certain action effect, so to speak.

Participant 06

### Quantitative Results

The One Button Tracker received a median grade of 7.5 (IQR 2.0) on a scale of 1 to 10, with individual grades ranging from 5 to 10. The SUS was completed by all participants. The median SUS score was 75.0 (IQR 17.50) out of a possible maximum score of 100, with individual scores ranging from 50.0 to 97.5. This corresponds to a percentile rank of 73% and indicates that the One Button Tracker’s usability can be considered as *good usability* [26,27].

## Discussion

### Principal Findings

In this study, we explored the potential of low-effort, in-the-moment self-tracking of subjectively experienced phenomena to support self-knowledge gain by focusing on one such option, the One Button Tracker.

All participants in this study successfully designed a personal research question in the context of a health promotion program and tracked one or more chosen phenomena with the device. The findings suggest that instrumentation options such as these can aid individuals in the pursuit of personal knowledge gain. However, such an approach may not suit everyone. Participants highlighted the user-friendliness of the One Button Tracker instrument; however, some barriers to swift data collection were also identified.

Participants generally considered the One Button Tracker as user-friendly, which was reflected by the median SUS score and grade the device received. However, technical issues encountered initially by some of the participants posed a risk to loss of the collected personal data, and with it, the instrument’s usability. Although such difficulties can occur in instruments that have not been tested extensively [15], problems of this type will have to be resolved through design testing before such self-tracking options are rolled out on a bigger scale [28,29]. Additional usability challenges associated with the physical design of the One Button Tracker, for instance, how to carry it when wearing clothes without pockets, were also a limitation.

Despite these barriers the One Button Tracker provided enhanced awareness and personal knowledge gain. Most participants felt using the One Button Tracker helped raise their awareness of the tracked phenomenon. They indicated that this awareness led to confirmation or gain of personal insights. This benefit was reported even though some participants experienced limited accuracy of the collected data caused by missed or misregistered observations. This suggests that high accuracy of the collected data may not be necessary to effectuate the positive effects, as almost all participants also reported instances of missed or misregistered data points. Further research is needed to evaluate how the accuracy of collected data might influence the quality of gained self-knowledge, a question that has been raised before [30], and how this quality might in turn influence users’ perceptions of health or health behavior.

Most participants experienced a change in health behavior, yet the measurability and sustainability of this change remained unclear. In line with the *self-improvement hypothesis of personal informatics*, participants believed that the increased awareness and gained personal knowledge resulting from the self-tracking process led them to change their behavior [31,32]. In addition, some participants felt the change in behavior was incentivized specifically by the sight of the instrument. Importantly, it is an experience of behavioral change that was assessed here; we did not measure actual change.

Participants emphasized the value of support during the different stages of the self-tracking process. The need for support is exemplified by the fact that most participants in present and previous studies struggled with interpreting the collected data on their own [20,21]. Furthermore, in line with previous research, regular interaction with the researchers was stated to be a motivating factor [4,32]. However, evidence that such support contributes to enhanced health effects as compared with self-tracking with no assistance is lacking [29,33,34]. Interview data indicate that the need for support was often related to help with the web-based data analytics tools.

### Implications

The diversity in participant views emphasizes the importance of providing an appropriate self-tracking option to each individual. It turned out that the low-effort in-the-moment approach is a good fit for some individuals, but not all. Previous research supports the notion that a one-size-fits-all approach does not exist; rather, different options should be developed for user groups with different needs [35-37]. Self-tracking can lead to negative effects such as feelings of failure and incompetence associated with individuals being reminded of the incongruence between their behavioral goal and the behavior actually performed [32,37-40]. Therefore, it is important to determine what does and does not work for different users or user groups.

The results found here have several implications for scientific research and medical practice. Academic researchers are becoming more aware of the contributions PLR can make to knowledge creation, such as generating hypotheses, enriching questionnaires, or answering questions through real-life data. However, before low-effort, in-the-moment tracking can be used as a scientifically sound data acquisition method, more clarity is needed on the quality of the collected data. In addition to the barriers described above, the tracked experience may also be distorted because it is being tracked, a phenomenon that is addressed as the *observer effect* [32,41]. Therefore, further research is needed into the validity and reliability of the collected data, and how these relate to those of other data collection methods. In a clinical context, it is clear that self-tracking of personally relevant phenomena may help some individuals gain self-insight and develop healthier behavior. However, for such methods to be successfully deployed in medical practice, it is important to gain a better understanding of the possible health effects, long-term effects, and potential differences in experiences between different user groups, as it has been shown previously that patients often differ in experiences from healthy volunteers [30,42]. This will help determine in which application areas self-tracking could be beneficial and how such a program could be best set up.

This study is among the first to explore this novel approach to self-tracking. An important strength of the study is that it provided participants with a chance to participate in a personalized form of PLR, in which they could set up and answer their own personally relevant research question. This provided a real-life setting, in which participants self-tracked for a purpose of their own choosing. In addition, the use of a mixed methods approach enhanced the strength of the findings, as the quantitative and qualitative findings converged well.

### Limitations

This study has several limitations. Data saturation was not reached. As a result, we cannot ensure that the entire range of possible participant experiences was covered. The participation rate was sufficient for saturation; however, it was relatively low (n=13). In our experimental setup, participants were allowed to track different types of phenomena, which explains the wide range of facilitators and barriers that were identified. Although this small group already provides valuable information in the context of an explorative study, a larger group may have revealed additional barriers and facilitators or may have provided a clearer image about the ones that stand out. Another limitation is related to the Hawthorne effect. Some participants found the research context itself to be motivating. This could mean that participants are less motivated to keep up a real-life self-tracking project. Finally, the participants included here were highly motivated, with an active participation in a health promotion program. This suggests that their experiences might not fully correspond with those of a population with less strong motivation or fewer support options.

### Concluding Remarks and Future Work

This study explored the potential usability and usefulness of low-effort, in-the-moment self-tracking for acquiring new personal insights and supporting changes in health behavior. Although the prototype self-tracking instrument studied here has shown itself to be perceived as user-friendly and can be used to quantify subjective experienced phenomena effectively, experiences in the participant group varied widely. Although the study has demonstrated the utility of the instrument in individual cases, the potential efficacy of the instrument in general is inconclusive at this point.

Before the One Button Tracker instrument can be provided to patients or study participants on a larger scale, the technical challenges and specific usability issues identified in this study should be addressed. In particular, usability issues related to wearability would need to be addressed.

As the study participants appreciated the support they received during the study, it would be interesting to study which kind of and what level of support is conducive for the process in the different steps of self-tracking as part of PLR or personal science. Further research is needed to assess the exact benefits of support and to evaluate how this support would best be provided. Here, the role and usability of the data analytics tool is also a topic for further research.

The explorative nature of this study and the overall purpose for using the One Button Tracker to track anything related to personal health and well-being has led participants to track a wide range of different phenomena. This demonstrated that participants can use the instrument in everyday life settings and acquire real-world data on subjective experience. Future studies could focus on addressing the application of the instrument in specific health domains to identify where self-tracking of subjective experience could potentially benefit diagnostics, health monitoring, or behavior change. For example, future studies could be conducted to understand to what extent the observed experiences would translate to objectively measurable change and how sustainable this change in behavior could be [14,31,32,43].

## Appendix

Multimedia Appendix 1 Overview with screenshots from the dashboard of the web-based analytics tool.

Multimedia Appendix 2 Interview guide (in Dutch).

Multimedia Appendix 3 Questions in the System Usability Scale (in Dutch).

## Abbreviations

PLR: participant-led research
SUS: System Usability Scale
