# Supplementary figures and images for: Barriers to and Facilitators of Using a One Button Tracker and Web-Based Data Analytics Tool for Personal Science: Exploratory Study
Source: JMIR Form Res. 2022 Mar 1;6(3):e32704. doi: 10.2196/32704 (PMC8924778; doi:10.2196/32704)

#
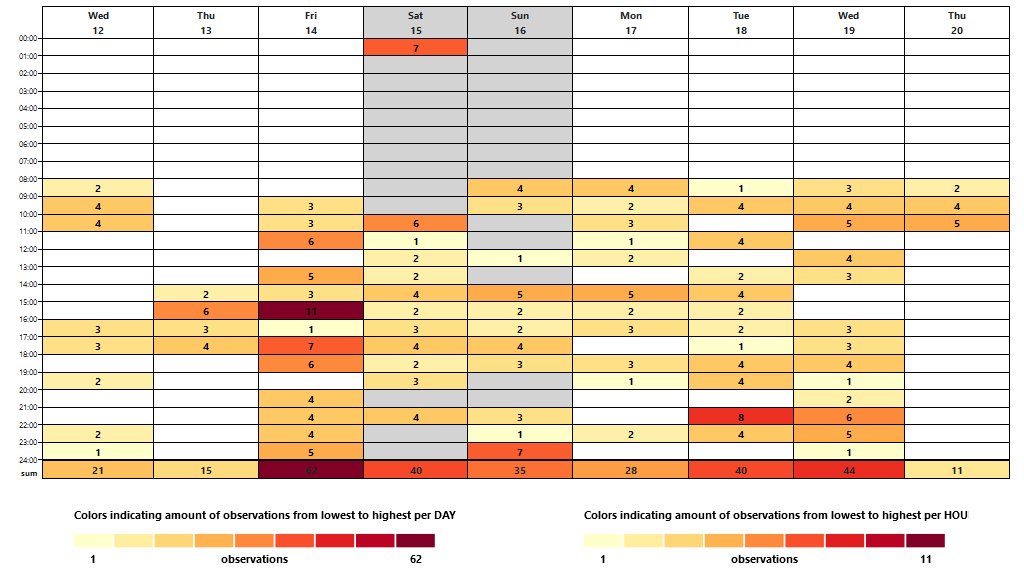


#
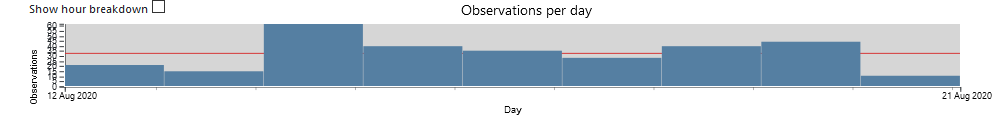

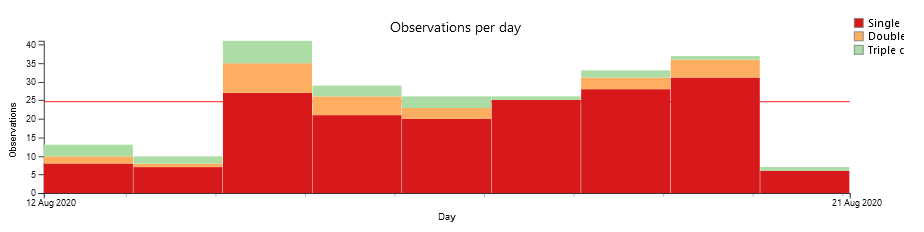

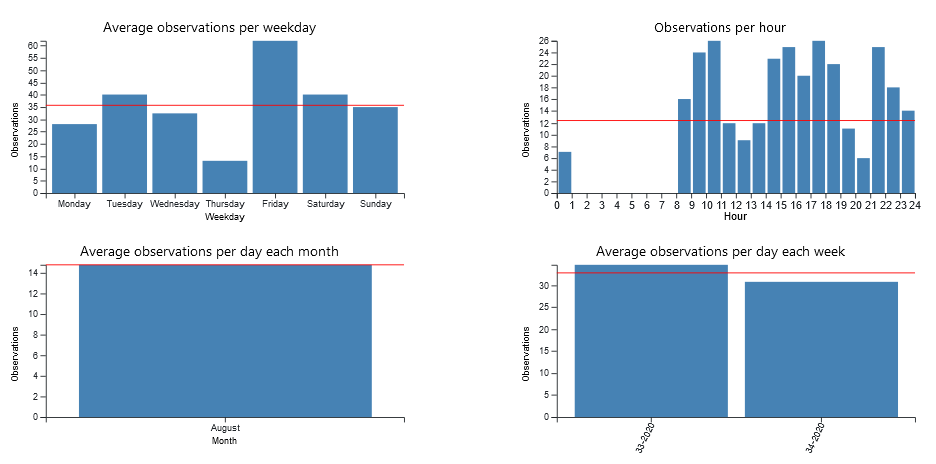

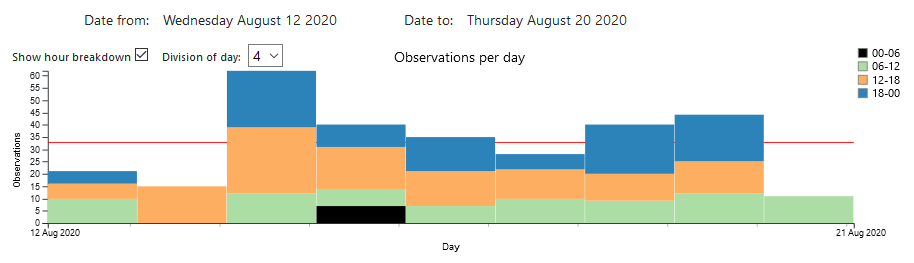


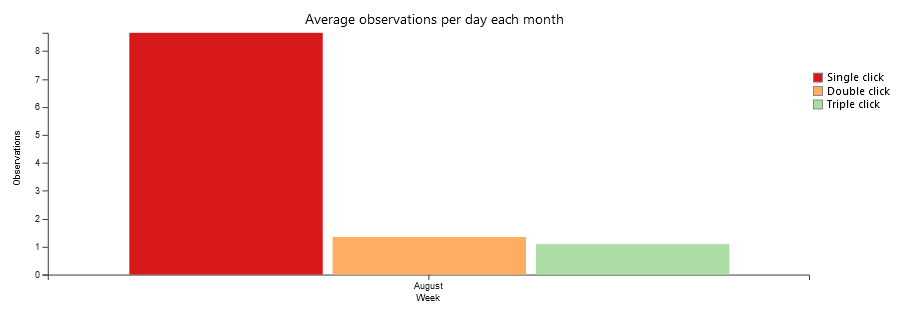

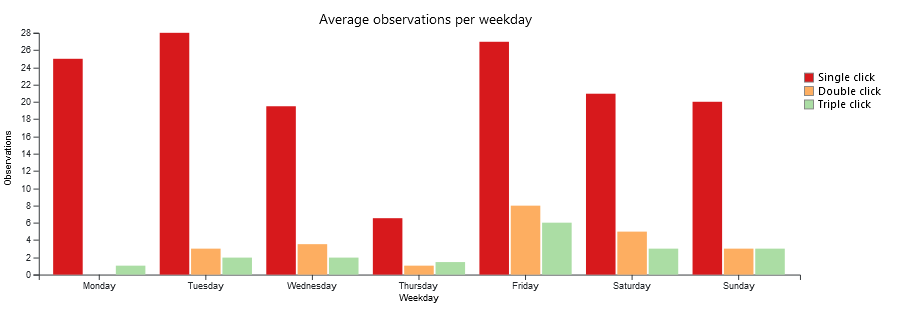

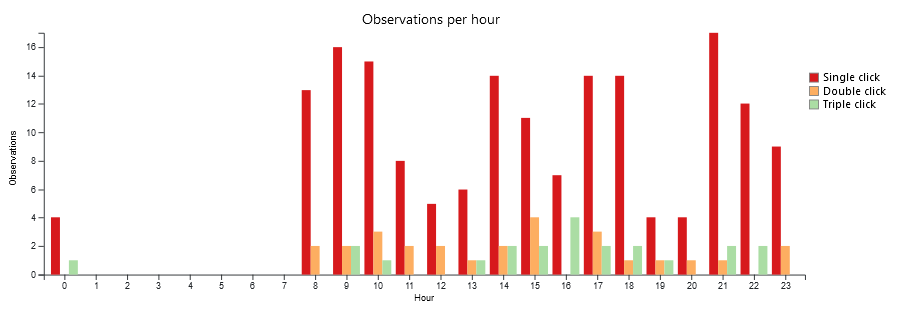

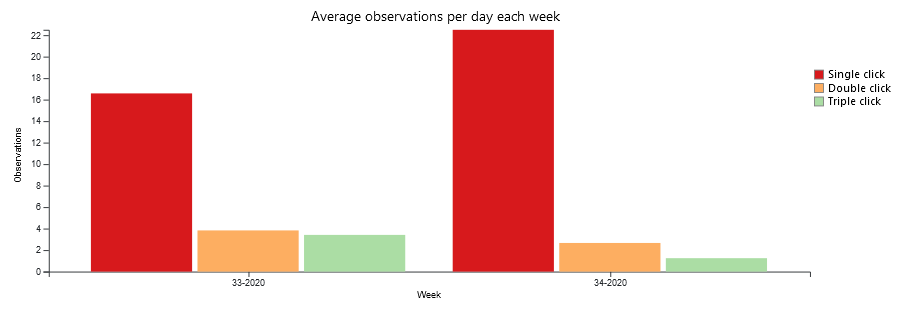

Supplement: Multimedia Appendix 1 [file formative_v6i3e32704_app1.docx]
