# Supplementary material for: Barriers to and Facilitators of Using a One Button Tracker and Web-Based Data Analytics Tool for Personal Science: Exploratory Study
Source: JMIR Form Res. 2022 Mar 1;6(3):e32704. doi: 10.2196/32704 (PMC8924778; doi:10.2196/32704)
Supplement: Multimedia Appendix 2 [file formative_v6i3e32704_app2.docx]

| Topics |
| --- |
| Verwachtingen  *Wat waren uw verwachtingen voorafgaand aan het meten?* |
| Onderzoeksvraag en bijgehouden variabele  *Met welke onderzoeksvraag bent u aan de slag gegaan? Welke waarde heeft u bijgehouden middels de OBT?* |
| OBT  *Hoe vond u het om de drukknop te gebruiken?* |
| Zelfmeten  *Hoe vond u het om iets van uzelf middels metingen bij te houden?* |
| Verzamelde data  *In hoeverre denkt u dat de verzamelde data overeenkomen met de werkelijke situatie?* |
| Dashboard  *Heeft u de data op het online dashboard bekeken? Wat vond u van het online dashboard?* |
| Gevolg van gebruik  *Hoe was het om de verzamelde data te bekijken? In hoeverre heeft u antwoord gekregen op de gestelde onderzoeksvraag?* |
| Omgeving  *In hoeverre wist uw omgeving dat u de drukknop gebruikte?* |
| Aanbevelingen  *Hoe zou u in de toekomst nogmaals iets bijhouden?* |
| Cijfer  *Welk cijfer zou u de OBT geven op een schaal van 0 tot 10?* |

*The subjects “omgeving” (environment) and “cijfer” (grade) were added after the pilot interviews.*
