# Supplementary material for: Barriers to and Facilitators of Using a One Button Tracker and Web-Based Data Analytics Tool for Personal Science: Exploratory Study
Source: JMIR Form Res. 2022 Mar 1;6(3):e32704. doi: 10.2196/32704 (PMC8924778; doi:10.2196/32704)
Supplement: Multimedia Appendix 3 [file formative_v6i3e32704_app3.docx]

1. **Ik denk dat ik het systeem vaak zou willen gebruiken**

**
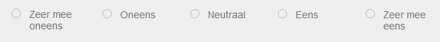
**

1. **Ik vond het systeem complex**

**
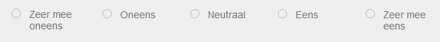
**

1. **Ik vond het systeem makkelijk om te gebruiken**

**
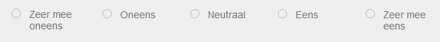
**

1. **Ik denk dat ik ondersteuning nodig heb om het systeem te kunnen gebruiken**

**
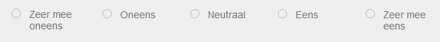
**

1. **Ik vond de verschillende onderdelen van het systeem samenhangend**

**
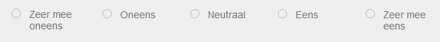
**

1. **Ik vond te veel inconsistentie in het systeem**

**
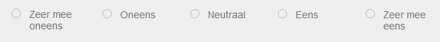
**

1. **Ik kan me voorstellen dat veel mensen op een snelle wijze het systeem leren gebruiken**

**
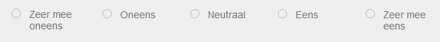
**

1. **Ik vond het systeem heel omslachtig om te gebruiken**

**
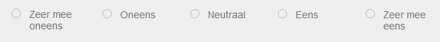
**

1. **Ik voelde me zeer vertrouwd bij het gebruik maken van het systeem**

**
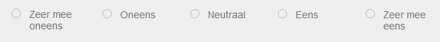
**

1. **Ik moest veel dingen leren voordat ik met het systeem aan de slag kon**

**
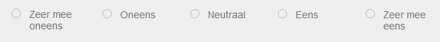
**
